# Large-scale and uniform preparation of pure-phase wurtzite GaAs NWs on non-crystalline substrates

**DOI:** 10.1186/1556-276X-7-632

**Published:** 2012-11-21

**Authors:** Ning Han, Jared J Hou, Fengyun Wang, SenPo Yip, Hao Lin, Ming Fang, Fei Xiu, Xiaoling Shi, TakFu Hung, Johnny C Ho

**Affiliations:** 1 Department of Physics and Materials Science, City University of Hong Kong, 83 Tat Chee Ave., Hong Kong, SAR 999077, China; 2 Centre for Functional Photonics (CFP), City University of Hong Kong, 83 Tat Chee Ave., Hong Kong, SAR 999077, China

**Keywords:** GaAs nanowires, Wurtzite phase, Non-crystalline substrates, P-type semiconductors, 61.46.Km, 73.63.Nm, 78.40.Fy

## Abstract

One of the challenges to prepare high-performance and uniform III-V semiconductor nanowires (NWs) is to control the crystal structure in large-scale. A mixed crystal phase is usually observed due to the small surface energy difference between the cubic zincblende (ZB) and hexagonal wurtzite (WZ) structures, especially on non-crystalline substrates. Here, utilizing Au film as thin as 0.1 nm as the catalyst, we successfully demonstrate the large-scale synthesis of pure-phase WZ GaAs NWs on amorphous SiO_2_/Si substrates. The obtained NWs are smooth, uniform with a high aspect ratio, and have a narrow diameter distribution of 9.5 ± 1.4 nm. The WZ structure is verified by crystallographic investigations, and the corresponding electronic bandgap is also determined to be approximately 1.62 eV by the reflectance measurement. The formation mechanism of WZ NWs is mainly attributed to the ultra-small NW diameter and the very narrow diameter distribution associated, where the WZ phase is more thermodynamically stable compared to the ZB structure. After configured as NW field-effect-transistors, a high *I*_ON_/*I*_OFF_ ratio of 10^4^ − 10^5^ is obtained, operating in the enhancement device mode. The preparation technology and good uniform performance here have illustrated a great promise for the large-scale synthesis of pure phase NWs for electronic and optical applications.

## Background

Due to the outstanding chemical and physical properties, III-V compound semiconductor nanowire (NW) materials such as InAs and GaAs are considered to be one of the most promising candidates for next-generation electronics and photonics [1-4]. Typically, they are synthesized *via* metal-catalyzed (e.g., Au, Ni) vapor–liquid-solid (VLS), and/or vapor-solid-solid (VSS) processes in metalorganic chemical vapor deposition (MOCVD) or molecular beam epitaxy (MBE) systems. During the growth, the temperature, V/III ratio, catalyst dimension, and other processing parameters can be precisely varied, aiming to control the crystal quality and growth orientation of NWs [5-9], in which the corresponding electrical and optical properties can then be tailored for various technological applications.

Since the cubic zincblende (ZB) crystal structure is more energy-stable than the hexagonal wurtzite (WZ) one in most of the bulk III-V semiconductors, the WZ structure is commonly observed as crystal defects (stacking faults and twin planes) in NWs due to the surface energy change in the nanometer scale [10,11]. Notably, pure WZ-structured NWs are only observed with small diameters (approximately 10 nm) [12]. These crystal defects are detrimental to electronic and optical properties of NWs; for example, the mixed phase InAs NWs have a resistivity up to 2 orders of magnitude higher than that of single phase NWs [13], and the electron mobility is found to decrease significantly in highly defective InAs and InP segments [14,15]. In general, the pure phase NWs can be prepared by controlling the basic growth parameters in MOCVD or MBE system, in which the low growth temperature and high V/III ratio favor the ZB structure, while the reverse condition is preferred for the WZ structure [5-7]. Even though the WZ structure dominates in the growth of thin NWs, the thick WZ ones can still be achieved by the lateral growth of firstly prepared thin WZ NWs with the tapering morphology and low defect density [12] at a high V/III ratio of 200. At the same time, single-crystalline wafers such as GaAs(111)B and InAs(111)B are usually used as substrates for the epitaxial growth of NWs. Nowadays, non-crystalline substrates are highly preferred as to lower the preparation cost and to ease the subsequent NW integration by assembling NW parallel arrays [16-18]. However, since Au/GaAs(111)B interface favors the WZ stacking while Au/GaAs(111)A interface favors the ZB structure [19], it is understood that it is even more difficult to achieve pure phase NWs on non-crystalline substrates, without the underlying lattice, which guides the formation of NWs. As a result, controlling the crystal structure of III-V NWs for the pure-phase and uniform property in the large scale is still a challenging topic.

Herein, in this work, pure-phase WZ GaAs NWs are successfully prepared on non-crystalline SiO_2_/Si substrates in the large scale by just controlling the NW diameter in the order of approximately 10 nm. XRD patterns show the first observation of pure WZ phase NWs grown on amorphous substrates in the literature, while the electronic bandgap determined by the reflectance spectra fits well with the relatively larger bandgap of WZ phase as compared to the one of ZB structure. Excellent electronic properties are also revealed after configuring the NW as a field-effect-transistor (FET).

## Methods

### Synthesis of GaAs NWs

A dual-zone horizontal tube furnace, one zone for the solid source (upstream) and one zone for the sample (downstream), was used as the reactor for the synthesis of GaAs NWs, as reported previously [17]. At first, thermal evaporation was carried out with 99.995% pure Au to deposit a 0.1-nm thick Au film on SiO_2_/Si substrates (50 nm thermally grown) under a vacuum of approximately 1 × 10^−6^ Torr. The processed substrate was then placed in the middle of the downstream zone with a tilt angle of approximately 20° and thermally annealed at 800°C for 10 min in a hydrogen environment to obtain Au nanoclusters as the catalysts. The solid source, GaAs powders (approximately 1.0 g), placed within a boron nitride crucible, was positioned in the upstream zone with a distance of 10 cm away from the sample. During the NW growth, the source was heated to the required source temperature (900°C), while the substrate was cooled to the preset growth temperature (580°C to 620°C). Hydrogen (99.9995% purity, 100 sccm) was used as the carrier gas to transport the thermally vaporized solid GaAs source to the downstream, and the pressure was maintained at approximately 0.5 Torr for the entire duration of the growth (1 h). After the growth, the source and substrate heater were stopped together and cooled down to room temperature under the hydrogen flow. In this case, the NWs were grown chemically intrinsic without any intentional dopants.

### Characterization of GaAs NWs

Surface morphologies of the grown GaAs NWs were examined with a scanning electron microscope (SEM, FEI Company, Oregon, USA/Philips XL30, Philips Electronics, Amsterdam, The Netherlands) and transmission electron microscope (TEM, Philips CM-20). Crystal structures were determined by collecting X-ray diffraction (XRD) patterns on a Philips powder diffractometer using Cu Kα radiation (*λ* = 1.5406 Å), imaging with a high resolution TEM (JEOL 2100F, JEOL Co., Ltd., Tokyo, Japan), and selected area electron diffraction (SAED, Philips CM-20). Elemental mappings were performed using an energy dispersive X-ray detector attached to the JEOL 2100F to measure the chemical composition of grown NWs. For the elemental mapping and TEM, the GaAs NWs were first suspended in the ethanol solution by ultrasonication and drop-casted onto the grid for the corresponding characterization. The reflectance spectrum was measured with a Lambda 750 spectrophotometer (PerkinElmer Inc., MA, USA) at room temperature.

The GaAs NWFETs were fabricated by drop-casting the NW suspension onto highly doped p-type Si substrates with a 50-nm thermally grown gate oxide. Photolithography was utilized to define the source and drain regions, and 50-nm thick Ni film was thermally deposited as the contact electrodes followed by a lift-off process. Electrical performance of fabricated back-gated FETs was characterized with a standard electrical probe station and Agilent 4155C semiconductor analyzer (Agilent Technologies, CA, USA).

## Results and discussion

**Figure 1:**
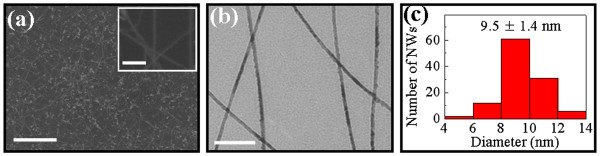
**SEM and TEM images depict the surface morphology of GaAs NWs.** (**a**) SEM image (scale bar = 2 μm) and magnified image in the inset (scale bar = 100 nm) and (**b**) TEM image (scale bar = 100 nm) of GaAs NWs grown by 0.1-nm thick Au film. (**c**) NW diameter distribution statistics performed in (b) is plotted after a measurement of approximately 100 NWs in TEM images.

**Figure 2:**
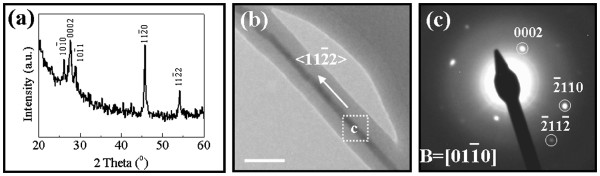
**XRD pattern of GaAs NWs and the TEM images of the corresponding SAED pattern.** (**a**) XRD pattern of GaAs NWs grown by 0.1-nm thick Au film on SiO_2_/Si substrates showing the wurtzite structure; (**b**) and (**c**) are the TEM images (scale bar = 50 nm) and the corresponding SAED pattern of one typical NW showing the WZ phase and growth orientation of <11 $\bar{2}$2>.

As shown in Figure 1, SEM and TEM images depict the surface morphology of GaAs NWs grown by the 0.1-nm thick Au catalyst film, where they all have a smooth surface, high aspect ratio, and uniform diameter along the entire length of NWs. The NW diameter distribution is carried out by measuring approximately 100 NWs in the TEM images, and the statistics shows an average diameter of 9.5 ± 1.4 nm. All of these have demonstrated a narrow diameter distribution utilizing this simple growth technique on non-crystalline substrates. Notably, the XRD pattern in Figure 2a agrees well with the WZ-structured GaAs (PDF 01-080-0003), which illustrates that the NWs were predominantly (>95% considering the resolution of XRD) grown in the pure WZ phase in the large scale. Furthermore, the SAED pattern of one typical NW (Figure 2b) is presented in Figure 2c, where the pattern also fits well with the hexagonal structure, and the specific NW is identified to grow in <11 $\bar{2}$2> direction.

**Figure 3:**
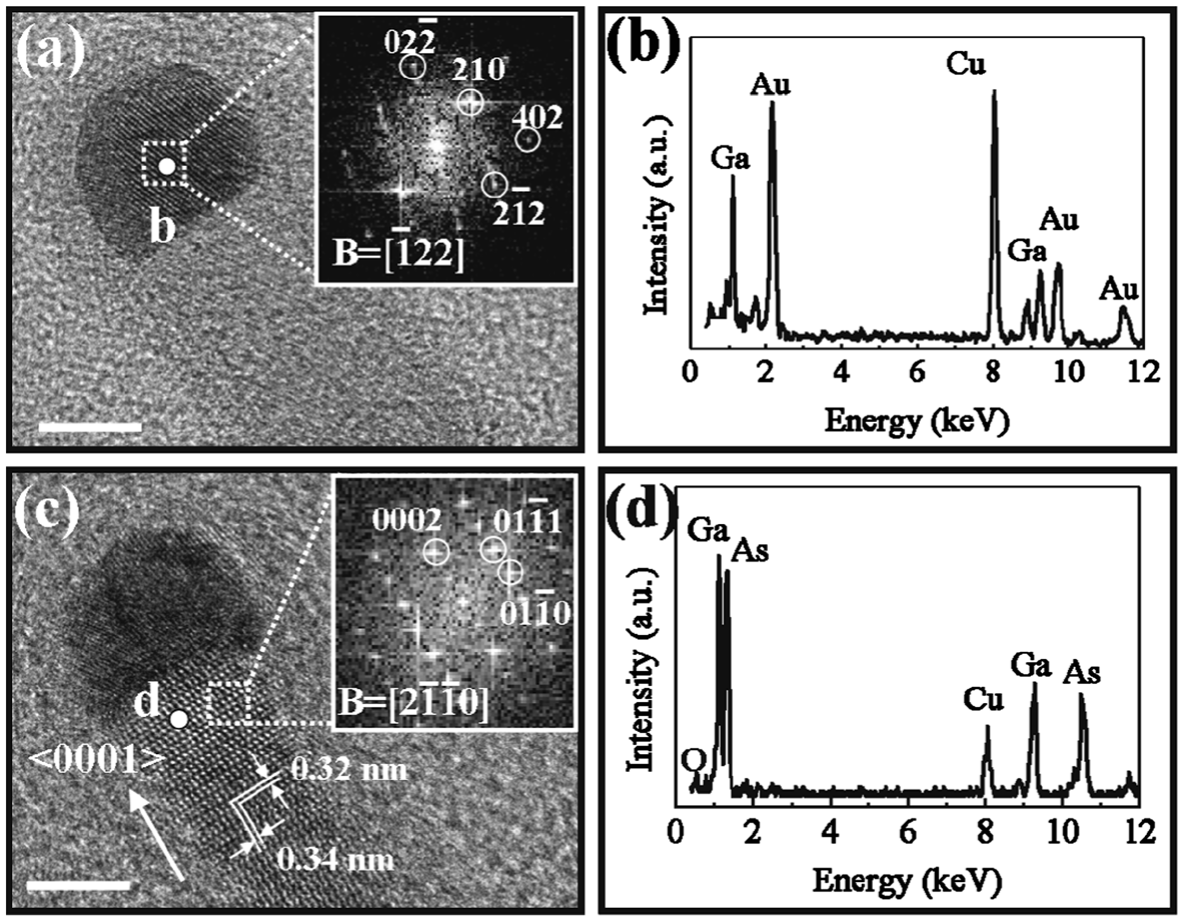
**HRTEM, the corresponding FFT images and EDS spectra.** (**a**) and (**c**) are the HRTEM images and the corresponding FFT (insets) of the catalyst tip and NW body (scale bar = 5 nm); (**b**) and (**d**) are the EDS spectra of the catalyst tip and NW body in (a) and (c), respectively. It is also noted that the FFT of NW body in (a) is shown in (c) as the tip and the body are not observed under the same zone axis.

To further study the crystal structure of grown GaAs NWs in detail, HRTEM images are obtained, and one typical NW is shown in Figure 3. A spherical catalytic tip is obviously observed in Figure 3a inferring a VLS/VSS growth mechanism of the GaAs NWs [8,20]. As verified by the EDS spectra (Figure 3b), the catalytic tip consists mainly of the Au and Ga constituents, with an atomic ratio approximating 1:1, and with neglectable concentration of As. This can be explained by the widely accepted VLS/VSS growth mechanism that Ga precursors come from the diffusion and precipitation of Au-Ga alloy tips, and As precursors are mainly provided from the ambient vapor, because of the low solubility of As in Au [8,20,21], reacting with Ga at the NW tip and body interface to yield NWs. The fast Fourier transform (FFT) of the Au-Ga alloy tip (Figure 3a inset) shows the crystal structure of the AuGa crystal viewed from $\bar{1}$22] direction, which well agrees with that of the standard AuGa alloy (PDF 3-065-1488); therefore, the GaAs NW is found catalyzed by the orthorhombic structured AuGa alloy, assuming no post-growth as the growth is stopped instantaneously with no Ga and As precursor supply. In the meanwhile, the HRTEM image in Figure 3c shows a good crystallinity of the NW body, with spacings of 0.32 and 0.34 nm, fitting well with those of {0002} and {10 $\bar{1}$0} planes, and the WZ structure is again verified by the FFT presented in the inset viewed from [2 $\bar{1}$$\bar{1}$0] direction. It is also noted that the growth direction of NWs is <0001> in Figure 3c, which is different from that of Figure 2b showing a mixed growth orientations in these NWs. Moreover, as indicated from the EDS spectrum (Figure 3d), the Ga/As ratio approximates 1:1, suggesting the stoichiometric composition of the grown NWs. Notably, a small amount of oxygen (approximately 5 atomic%) is also identified in the spectrum, which mainly comes from the amorphous surface oxide as shown in Figure 3c. The oxide surface (approximately 2 nm thick) would provide a high concentration of trap states in the interface of oxide/NW, which influences the electronic properties of GaAs NWs as we reported earlier [22], and will also be discussed in details in the following sections.

In this case, all the crystallographic studies have verified the WZ structure of GaAs NWs grown by 0.1-nm thick Au catalyst film deposited on non-crystalline substrates. Without the guidance of underlying crystalline lattice, the obtained pure WZ phase is mainly attributed to the ultra-small NW diameter and the very narrow distribution associated as shown in Figure 1c. It is commonly believed that the WZ phase is more preferred in small diameter III-V NWs due to the thermodynamically lower energy surfaces. Although the ZB phase is favored in the bulk material, when the materials are scaled down to the nanometer, there exists a transition regime where the dominant ZB phase is replaced by the WZ structure. NWs with the diameter of approximately 10 to 25 nm for GaAs and approximately 50 to 60 nm for InAs are reported as the critical diameters for this transition in the literature [11,12,23-25]. Consequently, as most of our NWs are grown in the diameter <14 nm (mean = 9.5 nm), well below the critical diameter, it is predictable that all NWs existed in the WZ phase on amorphous substrates.

**Figure 4:**
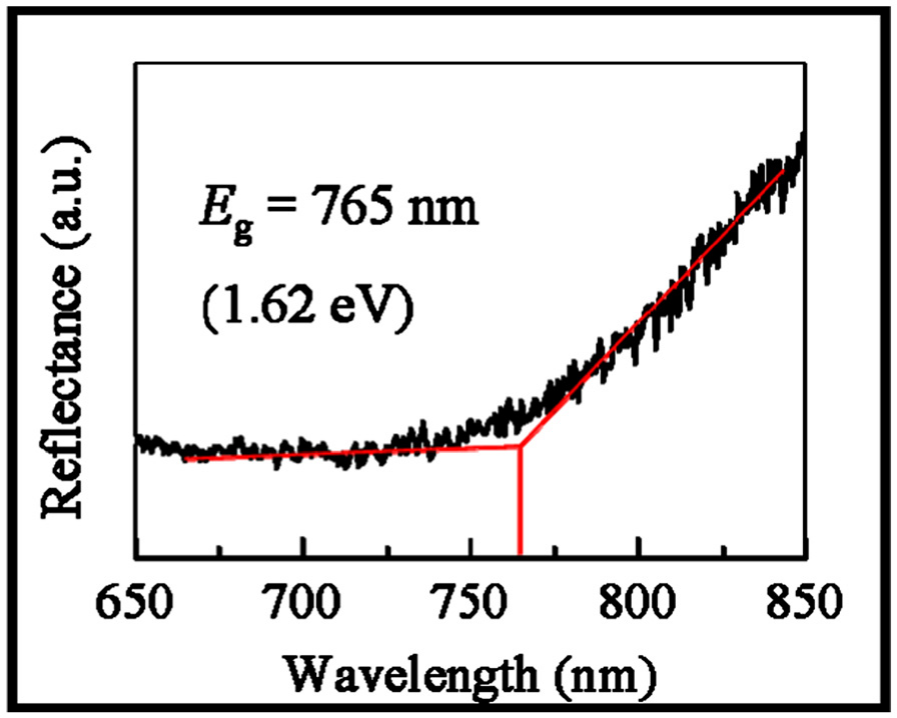
**Typical reflectance spectrum.** Reflectance spectrum (black line) of the GaAs NWs grown by 0.1-nm thick Au film on SiO_2_/Si substrates, which determines the electronic bandgap (*E*_g_) of approximately 1.62 eV by extrapolation (red line).

To shed light to explore the electronic bandgap of prepared GaAs NWs, reflectance measurement is performed, and the typical spectrum is shown in Figure 4. It is obvious that the incident light with a wavelength of <765 nm is mostly absorbed by GaAs NWs and thus has the lower reflectance intensity; this way, a bandgap (*E*_g_) of approximately 1.62 eV can be estimated. It is well accepted that the bandgap will be blue-shifted when the diameter of NWs is smaller than 20 nm, and a bandgap of 1.5 to 1.65 eV is usually determined in thin NWs in the literature [26-30]. As the bandgap of WZ GaAs is about 20 to 30 meV higher than that of the ZB GaAs [27,31], it is plausible that our WZ GaAs NWs have a bandgap of approximately 1.62 eV. It is also noted that no photoluminescence can be observed in our NWs which might be due to the trap states in the oxide/NW interface providing the recombination centers for the non-radioactive recombination of electron/holes pairs.

**Figure 5:**
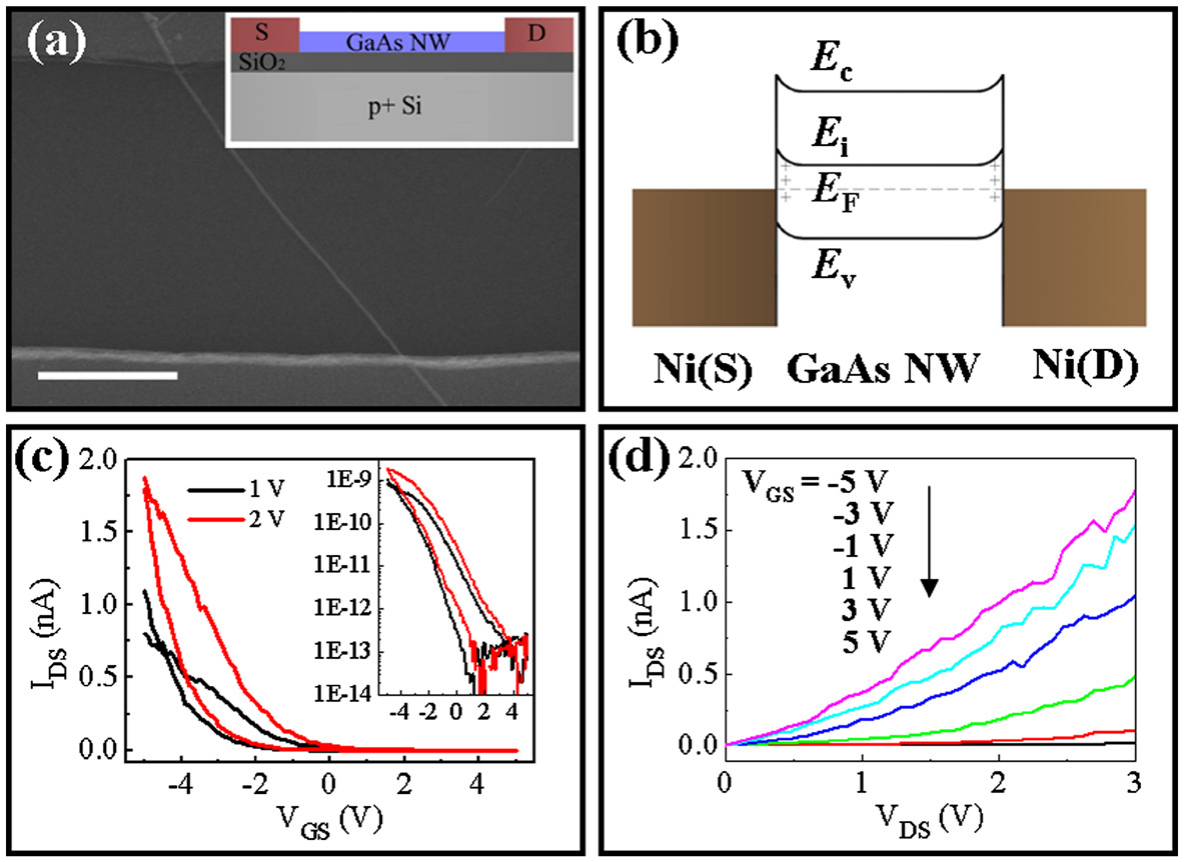
**SEM image, band diagram,*I***_**DS**_**-*V***_**GS**_**, and*I***_**DS**_**-*V***_**DS**_**curves.** (**a**) SEM image (scale bar = 1 μm) of one representative NW FET; the inset shows the schematic illustration of the back-gated FET configuration. (**b**) Band diagram of the NWs contacted with Ni source/drain electrodes. (**c**) *I*_DS_-*V*_GS_ curves and (**d**) *I*_DS_-*V*_DS_ curves of the NW FET.

At the same time, corresponding electrical properties are also investigated by fabricating the as-grown NWs into the back-gated FET, with one representative SEM image and the configuration scheme depicted in Figure 5a. As discussed above, there is a native oxide shell (approximately 2 nm thick) surrounding the GaAs NW, which provides electron trap states and then depletes the electrons in the GaAs NW core [32,33]. Typically for our unintentionally doped GaAs NWs, the ones with diameters below 40 nm would be fully depleted as to make a p-type semiconductor, while thick NWs are lightly depleted to persist the native n-type characteristics [22]. As a result, all the small-diameter WZ NWs exhibit typical p-type behaviors as shown in Figure 5c, where the *I*_DS_*V*_GS_ curves show the high ON/OFF current ratio (*I*_ON_/*I*_OFF_) of 10^4^ to 10^5^ operating in the enhancement mode, even though the ON current is relatively low in the magnitude of 10^−9^ A. The *I*_DS_*V*_DS_ curves in Figure 5d show a quasi-ohmic contact of the NW with Ni source/drain electrodes. In the ideal contact of Ni with clean GaAs surface, there should be a high Schottky barrier, theoretically considering the high work function of Ni approximately 5.2 eV and the low electron affinity of GaAs approximately 4.07 eV [34]. However, as the NW is fully depleted by the trapping states, the bands are all pushed upwards, and the oxide shell outside the GaAs NW would pin the Fermi level (*E*_F_) at approximately 1/3 *E*_g_ above valence band (*E*_v_) regardless of the metal used [35], as shown in the band diagram (Figure 5b). Consequently, the Schottky barrier becomes significantly smaller and thus makes the Ni-GaAs NW quasi-ohmic contacts.

## Conclusions

In summary, the high aspect ratio, smooth, large-scale, and uniform WZ GaAs NWs are prepared on non-crystalline SiO_2_/Si substrate, utilizing 0.1-nm thick Au film as the catalyst. The WZ structure is verified by specific characteristics in the XRD pattern, SAED pattern, HRTEM, and FFT. The electronic bandgap is also determined to be approximately 1.62 eV by the reflectance measurement. Notably, the WZ GaAs NWs all exhibit p-type semiconducting behavior with a high *I*_ON_/*I*_OFF_ ratio of 10^4^ to 10^5^, as revealed from the electrical characterization in fabricated back-gated NWFETs. All of these have demonstrated the successful control of pure WZ NWs grown on non-crystalline substrates, which present the potency of large-scale preparation for various high performance technological applications.

## Competing interests

The authors declare that they have no competing interests.

## Authors’ contributions

NH prepared the NWs and drafted the manuscript. JJH fabricated the NWFET, and SY made the I-V measurement. FW made the SEM and TEM observations, and HL calculated the NW diameter distribution. MF carried out the XRD measurement, and FX made the SAED identification. XS carried out the reflectance spectrum, and TH observed the HRTEM and EDS. JCH provided the idea and completed the manuscript. All authors read and approved the final manuscript.
